# Status and access to the Collaborative Cross population

**DOI:** 10.1007/s00335-012-9410-6

**Published:** 2012-07-31

**Authors:** Catherine E. Welsh, Darla R. Miller, Kenneth F. Manly, Jeremy Wang, Leonard McMillan, Grant Morahan, Richard Mott, Fuad A. Iraqi, David W. Threadgill, Fernando Pardo-Manuel de Villena

**Affiliations:** 1 Department of Computer Science, Lineberger Comprehensive Cancer Center, University of North Carolina at Chapel Hill, Chapel Hill, NC 27599 USA; 2 Department of Genetics, Lineberger Comprehensive Cancer Center, University of North Carolina at Chapel Hill, Chapel Hill, NC 27599 USA; 3 The Western Australian Institute for Medical Research and Centre for Medical Research, University of Western Australia, Perth, WA Australia; 4 Wellcome Trust Centre for Human Genetics, University of Oxford, Oxford, OX3 7BN UK; 5 Department of Human Microbiology, Tel Aviv University, Ramat Aviv, 69978 Tel Aviv, Israel; 6 Department of Genetics, North Carolina State University, Raleigh, NC 27695 USA

## Abstract

The Collaborative Cross (CC) is a panel of recombinant inbred lines derived from eight genetically diverse laboratory inbred strains. Recently, the genetic architecture of the CC population was reported based on the genotype of a single male per line, and other publications reported incompletely inbred CC mice that have been used to map a variety of traits. The three breeding sites, in the US, Israel, and Australia, are actively collaborating to accelerate the inbreeding process through marker-assisted inbreeding and to expedite community access of CC lines deemed to have reached defined thresholds of inbreeding. Plans are now being developed to provide access to this novel genetic reference population through distribution centers. Here we provide a description of the distribution efforts by the University of North Carolina Systems Genetics Core, Tel Aviv University, Israel and the University of Western Australia.

## Introduction

The Collaborative Cross (CC) is a mouse genetic reference population conceived 10 years ago as a community resource for systems genetics (Threadgill and Churchill 2012; Threadgill et al. 2002). It is a panel of recombinant inbred (RI) lines generated by randomly mixing the genetic diversity of eight extant inbred mouse lines: A/J, C57BL/6J, 129S1/SvImJ, NOD/ShiLtJ, NZO/H1LtJ, CAST/EiJ, PWK/PhJ, and WSB/EiJ. The genetic architecture of the CC population has been recently reported (Collaborative Cross Consortium 2012).

The CC was started with mice from The Jackson Laboratory in three separate locations: the Oak Ridge National Laboratory in Tennessee (Chesler et al. 2008), whose population was moved to The University of North Carolina at Chapel Hill; The International Research Livestock Institute in Kenya, whose population was moved to Tel Aviv University in Israel (Iraqi et al. 2008); and Western Australian Institute for Medical Research/University of Western Australia/Geniad, Ltd in Perth (Morahan et al. 2008). All CC lines were independently bred in a similar funnel breeding scheme that combined the genetic variation present in the eight founders into CC lines over three generations followed by inbreeding to homozygosity (Churchill et al. 2004).

In the past years an increasing number of reports have established the value of the CC to provide insights in the genetic architecture of multiple traits, the identification of novel loci associated with them, and the characterization of functional novel alleles at known genes (Aylor et al. 2011; Bottomly et al. 2012; Durrant et al. 2011; Kelada et al. 2012; Mathes et al. 2010; Philip et al. 2011).

## Distribution of the CC lines

The CC population will be maintained and distributed by distribution centers that reflect the needs of the research community and of the institutions involved in the generation of the cross. Currently, independent sets of CC lines are being generated at Chapel Hill in the US (CC-UNC), Tel Aviv in Israel (CC-TAU), and Perth in Australia (CC-GND) (Collaborative Cross Consortium 2012).

Eventually, distribution centers will have stocks of each CC line from the three production centers. However, as of the writing of this report, only the Chapel Hill site has breeders from all three populations (CC-UNC, CC-TAU, and CC-GND). There are about 130 CC lines (CC-TAU), between the 13th and 29th generations of inbreeding that are under development at Tel Aviv University and maintained in a conventional mouse facility. There are about 180 lines under development up to the 26th generation of inbreeding in Australia. These lines will be available for the research community as they reach the milestones for inbreeding discussed below. CC-TAU and CC-GND lines are under rederivation to specific-pathogen-free (SPF) condition at UNC. The rederived CC-TAU and CC-UNC lines will be shipped to TAU for maintenance in their SPF facility. This current state reflects the fact that UNC is equipped and funded to use dense genotyping to accelerate inbreeding through marker-assisted inbreeding (MAI) in order to expedite access to all CC lines (Collaborative Cross Consortium 2012; Welsh and McMillan 2012). The following sections provide a brief overview of the UNC Systems Genetics Core (http://csbio.unc.edu/CCstatus/index.py), using a structure that each distribution center has agreed upon to standardize operating procedures to ensure long-term genetic integrity of the CC population. Given that TAU has recently become a European Mouse Mutant Archive (EMMA) node and is therefore fully equipped to manage the colony, it is expected that it will be a key part in the distribution of CC in Europe. CC-GND strains have been shipped to UNC and it is envisaged that Geniad will manage distribution in Australia and Asia. Distribution centers will provide CC mice on a cost recovery basis such that the price per mouse should be similar to those of suppliers of genetically defined stocks (e.g., The Jackson Laboratory).

## Criteria for distribution

Historically, RI lines have been deemed inbred after 20 generations of brother-sister mating, and lines within a given panel have been made available as they reached this milestone. The development of genotyping arrays for the mouse (Collaborative Cross Consortium 2012; Yang et al. 2009) has made it possible to substitute this arbitrary threshold with an objective genetic criteria, namely, the level of residual heterozygosity in a given line. After consultation with the External Advisory Board of the UNC CC effort (Collaborative Cross Consortium 2012), and after discussion with investigators leading the CC-TAU and CC-GND breeding programs, we decided that lines that reach specific levels of inbreeding should be made available to the public:

- Lines are declared *complete* once they have reached 98 % homozygosity.
- Lines are declared *distributable* once they have reached 90 % homozygosity.

The first threshold is informed by the observation that many commonly used inbred lines have similar levels of residual heterozygosity (Yang et al. 2011; FPMV unpublished). The need for the second more relaxed criterion reflects our goal to accelerate access by all investigators to the CC resource. We also expect that even incipient inbred lines will be a powerful genetic resource. Additionally, it addresses the fact that some lines become increasingly difficult to maintain with terminal inbreeding and thus may be lost. Researchers need to carefully evaluate whether completed and/or distributable CC lines meet the needs of their experimental design. However, we wish to note that for decades geneticists have used resources with similar shortcomings and that in contrast with these historical resources, users of CC lines will know, with an unprecedented level of detail, which regions are not fixed and what alleles are segregating in these regions (see below for discussion on genotypes).

## Genome of CC lines

Measuring heterozygosity in CC lines requires dense genotyping of key mice in each line using a cost-effective genotyping platform coupled with sophisticated methods for haplotype reconstruction. Currently we are using the Mouse Universal Genotyping Array (MUGA) (Collaborative Cross Consortium 2012) developed at UNC to guide MAI in the CC population. The array has 7,500 informative SNPs and has been critical for projects using the CC and DO populations (Collaborative Cross Consortium 2012; Svenson et al. 2012).

**Fig. 1:**
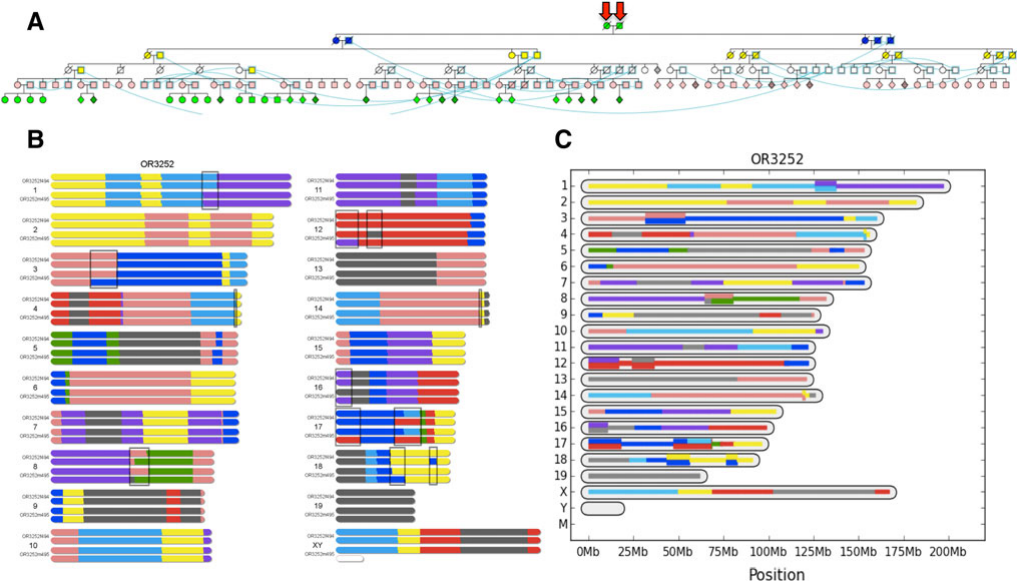
**a** Partial view of the pedigree of the OR3252 CC line. Mice are represented using standard symbols for human pedigrees. Mice that are present multiple times (because they participate in multiple matings) are linked by *blue curved lines*. *Colors* represent different generations of inbreeding. Mice shown at the top of the pedigree with *arrowheads* are the obligate ancestors of this line used to determine whether it passes the threshold for distribution (most recent obligate ancestors). **b** Genome of obligate ancestors based on MUGA genotypes. We use standard *colors* and a single-letter code to represent the contribution of the eight CC parental strains (Collaborative Cross Consortium 2012) to the genome of the two most recent obligate ancestors. Briefly, A/J, A, *yellow*; C57BL/6J, B, *gray*; 129S1/SvImJ, C, *pink*; NOD/ShiLtJ, D, *dark blue*; NZO/H1LtJ, E, *light blue*; CAST/EiJ, F, *green*; PWK/PhJ, G, *red*; and WSB/EiJ, H, *purple*. The two autosomes and the corresponding complement of X chromosomes for each mouse are drawn to illustrate the regions that are fixed (all four autosomes or three X chromosomes have the same haplotype) or segregating (shown in *boxes*). **c** The genome of the OR3252 line. The figure represents fixed regions as *single lines* and segregating regions as *double lines* of the appropriate colors

A key step in determining whether a line has reached distributable or completed status is the identification of obligate ancestors in the line of all extant mice or subsets of extant mice with limited heterozygosity (Fig. 1). We modified the CC database CCDB (Chesler et al. 2008) to generate pedigree reports using the Cranefoot program (http://www.finndiane.fi/software/cranefoot/) (Mäkinen et al. 2005). Cranefoot displays allow easy viewing of each CC line at any generation, showing branches or arms in the pedigree (Fig. 1a). In addition, a custom Python script, mrca.py, determines obligate ancestors for any subset of the current generation of mice.

**Fig. 2:**
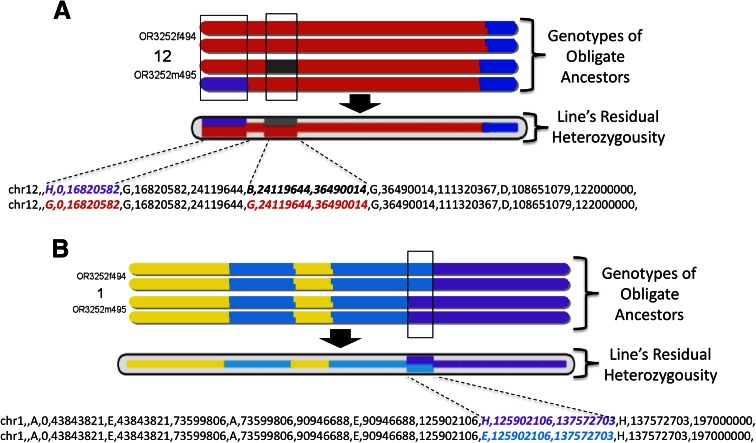
Residual heterozygosity in distributable lines. The figure shows two chromosomes from line OR3252 (shown in Fig. 1) to illustrate the identification of segregating regions in distributable lines. The figure follows the conventions detailed in Fig. 1, with the top part of each subheading representing the contribution of the eight CC parental strains to the genome of the two most recent obligate ancestors and the midsection and lower sections representing the haplotypes of the line as provided in the CC website as figure or as text, respectively. **a** Chromosome 12 illustrates two segregating regions in which one of the most recent ancestors is homozygous while the other is segregating. The figure also illustrates that in some cases the most recent obligate ancestors may appear to have slightly different boundaries between parental contributions. We suggest that investigators rely on the haplotype reconstruction provided in the text file rather than on visual inspection of most recent ancestors. We expect these discrepancies to be resolved in the near future with use of MegaMUGA. **b** Chromosome 1 illustrates a segregating region in which each of the most recent ancestors’ parents was homozygous for a different parental allele

Genotypes from MUGA are used for haplotype reconstruction as described previously (Collaborative Cross Consortium 2012). The haplotype reconstructions of the obligate ancestors are jointly considered to determine the maximum heterozygosity of a distributable line (Fig. 1b). Calculating the joint heterozygosity involves two steps: establishing recombination breakpoints and determining segregating regions within and between the obligate ancestors (Fig. 2). Recombination breakpoints are estimated by the midpoint of any ambiguous region found by the haplotype reconstruction (these tend to be no more than 2–3 SNPs). Ambiguous heterozygous regions within an ancestor begin and end at the closest heterozygous genotype call. Ambiguous heterozygous regions between ancestors begin and end at the nearest informative genotype. When genotype calls are consistently inconsistent with the intensity-based founder assignment (Collaborative Cross Consortium 2012), we assume that this is a feature of the line’s haplotype and treat the region as fixed. We then compute the genomic length of all segregating regions divided by the full genomic length to determine the maximum residual heterozygosity within a line. If lines have reached the required thresholds, we generate a special haplotype file for the entire distributable line indicating regions fixed for a specific CC founder and regions that are still segregating and between which CC founders they are segregating (Fig. 1c). All computations are based on Mb distances of the NCBI m37 version of the mouse assembly.

**Fig. 3:**
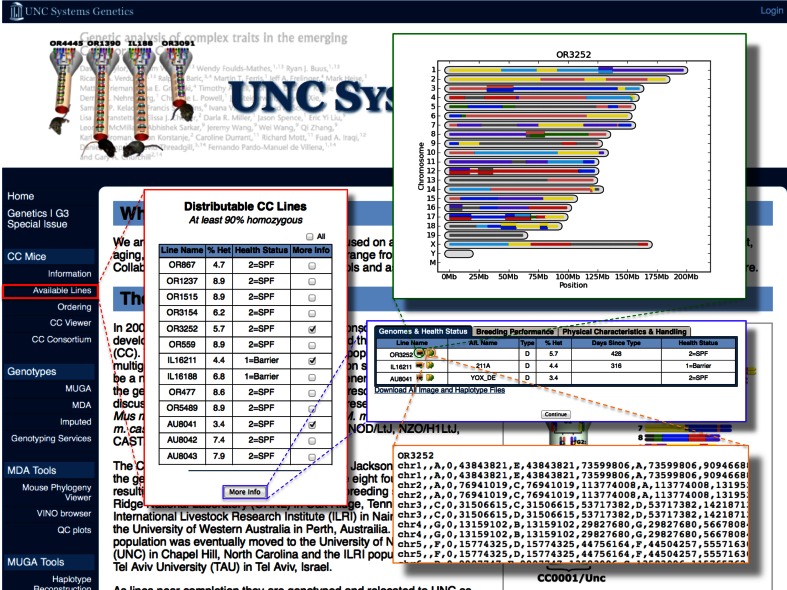
The UNC Systems Genetics Core web site. Screenshots of the main pages associated with the distribution of CC lines are shown. The “Available Lines” tab is highlighted on the *left side* of the web site as well as inserts of the pages associated with information on the number, genome, and characteristics of the available CC lines. Links from the menu take you to the ordering page and the CC viewer

**Fig. 4:**
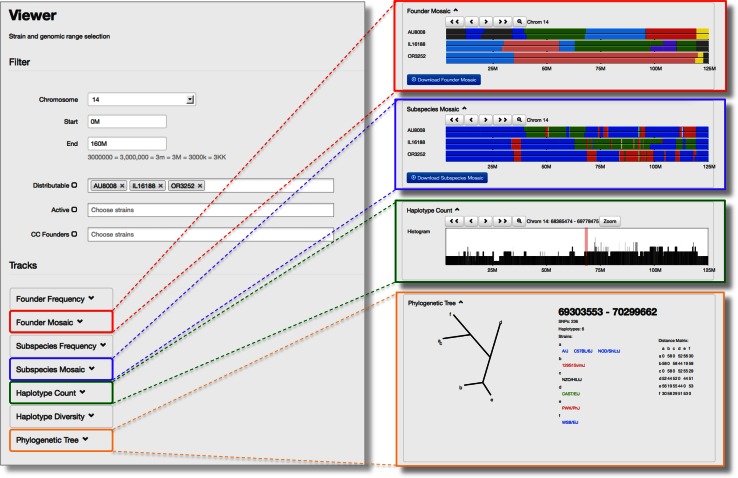
The CC Viewer allows comparative analysis and visualization of multiple collinear genomes (Wang et al. 2012b) and follows the conventions reported previously (Collaborative Cross Consortium 2012; Yang et al. 2011). On the *left side* is a screenshot of the CC Viewer web site showing the fields for selection of the genomic region (chromosome, start and end), type, and identity of CC line(s) to be viewed. Note that in addition to the distributable lines, active lines, and CC founders, the browser has data for the entire CC population reported previously (Collaborative Cross Consortium 2012). On the right side of the figure is the output for four of the seven possible tracks for three distributable lines for chromosome 14. Data underlying the “Founder Mosaic” and “Subspecific Origin” tracks can be downloaded by clicking the appropriate blue tab. The phylogenetic tree for a specific region can be selected by clicking on the appropriate chromosome location in the “Haplotype Count” track. Users can zoom in by selecting a region in any of the tracks or by using the zoom tab. Clicking also allows centering the view on a given region and sorting according to the type of data shown in that track

The haplotype assignments for each line can be visualized (as shown in Fig. 1c) or downloaded as text files (as shown in Figs. 2 and 3) from the UNC Systems Genetics Core web site (Fig. 3). The haplotype can also be visualized at http://www.csbio.unc.edu/CCstatus/index.py?run=CCV with the updated version of the CC Viewer (Fig. 4) (Collaborative Cross Consortium 2012).

Because in most cases several generations separate the obligate ancestors from the mice accessible to researchers, residual heterozygosity is overestimated (i.e., distributed mice are more inbred than advertised). On the other hand, currently we do not track the mitochondrial genome or Chromosome Y due to the lack of informative SNPs in MUGA. Furthermore, the SNP density in MUGA has limits of detection that will miss very short haplotype segments, especially in the telomeric ends of chromosomes (Collaborative Cross Consortium 2012). To address these limitations we will repeat the procedure with new obligate ancestors from each CC line as the breeding progresses. We also plan to repeat the procedure with a new genotyping array that contains ten times more SNPs that will be available in the summer of 2012 (MegaMUGA).

## Health status

At UNC, the distributable and completed CC lines have one of two health statuses, both of which are specific-pathogen-free (SPF) or cleaner (Threadgill et al. 2011). The SPF facility is designated health status “2” and is negative for all of the following by serology: EDIM, TMEV GDVII, MHV, *Mycoplasma pulmonis*, MPV, MVM, Parvo NS-1, PVM, and Sendai. Additionally, some are tested for CAR bacillus, Ectromelia, LCMV, MAD1, MAD2, mCMV, Polyoma, and REO3. The barrier facility is designated health status “1” and is negative for all of the above PLUS the following by serology: MNV, some are tested for PVM; by culture nasal swab: *Pasteurella pneumotropica*; and by fecal PCR: *Helicobacter*. The different health statuses of the lines in the UNC Systems Genetics Core reflect their different origins and our efforts to rederive distributable and completed CC lines through IVF and C-section to the clean barrier “1” facility.

Currently at TAU, all CC mice are maintained in a conventional facility, while the rederived lines will be maintained in individual ventilation cages (IVC) and housed at the newly established SPF facility. Mice will be tested for routine microbiology monitoring according to Federation of European Laboratory Animal Science Association (FELASA) recommendations (Nicklas et al. 2002).

## Access to CC lines, genotypes, and breeding information

All distributed CC animals fall under a Conditions Of Use (COU) that is virtually identical to the COU from The Jackson Laboratory. In essence, CC mice are available to any institution for internal use and cannot be redistributed to any researchers from other institutions without prior permission. Please note that all mice are provided as is and without liability to the provider.

We are striving to make all genetic information on CC lines available through a dedicated web site: http://csbio.unc.edu/CCstatus/index.py. The menu bar offers a link to the CC resource (“CC Mice”) and specific pages for information on “Available Lines,” “Ordering,” and the “CC Viewer” (Fig. 2). The “Available Lines” page lists CC lines that are in the Distributable and Completed categories, the percentage of the genome still known to be segregating, and the health status of the facility from which these mice are available. Researchers can obtain further information on the genotypes, breeding performance, physical characteristics, and handling information for any of these lines by checking “More Info” and using the corresponding tab. Importantly, this information includes the number of days since the birth of the obligate ancestors used to estimate maximum residual heterozygosity. This can be used to estimate additional inbreeding that has occurred in any line at a given date. Since three wild-derived strains were included in the original eight founder strains, some strains still exhibit jumpy characteristics and extra caution in handling should be observed.

CC mice can be ordered using the “Ordering” tab. Interested parties must register on the web site before ordering mice. By clicking on the “Ordering” tab, the COU, as previously mentioned, must be agreed to before proceeding. Data collection forms appear asking for pertinent shipping and ordering information.

A custom genome browser for the CC has been described previously (Collaborative Cross Consortium 2012). This CC Viewer has been updated to include the genome of all CC lines in the distribution center (Fig. 3). Users can visualize the genome of selected CC lines based on location and use interactive tools to zoom in and out, center, and order these lines as desired. In addition to the haplotype reconstruction, the site provides the subspecific origin based on diagnostic SNPs (Yang et al. 2011) and a tree reflecting the phylogeny of the founder strains at any given location. Data can be downloaded for further analysis.

## Future developments

There are currently 42 CC lines listed in the UNC System Genetics Core as distributable or complete, but we expect a rapid increase in number during 2012 (>50 lines distributable) and 2013 (>100 lines distributable). We also expect to provide an ever more accurate picture of their genomes through additional genotyping using the new MegaMUGA array. We suggest that interested parties check on availability and further developments such as the ability to download complete genome sequences for each CC line generated by imputations of the SNPs and indels found in each CC founder strain at the corresponding location of the genome. Pseudogenomes will be based on imputation of sequence variants found in the eight founders through whole-genome sequencing (Keane et al. 2011; Wang et al. 2012a). These pseudogenomes (provided in FASTA format) will be especially useful for investigators using NGS methods such as RNAseq.

Tel Aviv University, Geniad, Ltd in Perth, and The Jackson Laboratory are actively seeking the establishment of additional distribution centers. Finally, we are keenly aware of the value and the effort invested in the CC resource. To preserve the resource for the future, there are active efforts to archive embryos of completed and distributable CC lines at the UNC Mutant Mouse Regional Resource Center, the Wellcome Trust, and EMMA consortium.
